# *Salmonella* Control Programme in France: Factors Influencing the Detection of *Salmonella* in Laying Hen Flocks From 2013 to 2021

**DOI:** 10.1111/zph.70001

**Published:** 2025-07-16

**Authors:** Adeline Huneau‐Salaün, Sophie Le Bouquin, Marianne Chemaly

**Affiliations:** ^1^ ANSES ‐ Laboratoire de Ploufragan‐Plouzané‐Niort Ploufragan France

**Keywords:** epidemiology, France, hen, *salmonella*

## Abstract

**Introduction:**

Salmonellosis is the second leading foodborne illness in the European Union. Eggs are still an important source of *Salmonella* despite an EU‐harmonised control programme in laying hen flocks. The objective of our study was to identify the characteristics related to poultry house (location, type of housing system) and sampling (sampler, type and number of samples, date) associated with the detection of *Salmonella* target serovars (STS) in France ( *S. enteritidis* , *S. typhimurium* , three monophasic variants of *S. typhimurium* and *S*. Kentucky).

**Methods:**

For the first time since the implementation of the EU target prevalence in 2010, we compiled the results of bacteriological detection of *Salmonella* in French laying hen flocks (108,718 sampling events carried out in 4744 poultry houses). The risk of STS detection was modelled using a mixed logistic regression model taking into account repeated sampling at the poultry house level.

**Results:**

An STS was isolated from 737 sampling events (0.68%). Caged flocks had a higher risk (odds ratio (OR) = 1.6, 95% confidence interval [1.2–2.0]) of testing positive compared with on‐floor, organic or free‐range flocks. The risk of detecting STS was higher when sampling was carried out by the competent authority (CA) (OR = 2.62, [2.2–3.1]) relative to food business operators (FBO), in relation to the risk‐based sampling strategy used by the CA. A higher risk of STS detection was associated with taking six samples or more per sampling (OR = 2.8 [2.0–4.0]). A spatial gradient of risk was also described, running from the north‐west to the south‐east regions of France, in addition to seasonal (third quarter of the year: 2.8 [2.2–3.5], fourth quarter: 2.4 [1.9–3.0], relative to the first quarter) and annual effects (2016: 1.7 [1.2–2.5], 2020: 2.1 [1.5–2.9], 2021: 2.0 [1.4–2.8], relative to 2013).

**Conclusions:**

Our findings are of interest for improving sampling protocols for *Salmonella* detection in laying hen farms.

**Summary**

- The study presents the first results of the *Salmonella* national control programme (NCP) in laying hen farms in France from 2013 to 2021. France is one of the leading egg producers in the European Union (EU). Adopted in 1998, the French NCP was modified in 2013 to comply with EU regulations, but results have never been compiled before.
- The proportion of sampling events identifying a *Salmonella* target serovar varied from 0.48% in 2013 to 0.99% in 2020. The risk of *Salmonella* detection is higher during the last two quarters of the year, in eastern and southern France, in cage poultry houses relative to on‐floor, organic and free‐range houses, when sampling is performed by the competent authority and when more than 5 samples are taken.
- Improvements of the data collection system at the flock level are needed to better evaluate the impact of NCP on *Salmonella* infection in laying hen flocks in response to an increasing proportion of contaminated farms.

## 1 Introduction

Salmonellosis is the second leading foodborne gastrointestinal infection reported in the European Union (EU) with 77,486 cases in 2023 (EFSA and ECDC 2024). Congruent results from foodborne outbreak investigations and source attribution models indicate eggs and egg products as a major source of *Salmonella* infections in humans, although other sources such as pork products or other meat products also play a role in the *Salmonella* burden in the EU (EFSA 2019; Chaname Pinedo et al. 2022). Eggs were the first food vehicles implicated in salmonellosis foodborne outbreaks in the EU in 2023 (EFSA and ECDC 2024): they were involved in 32% of the strong‐evidence outbreaks, followed by mixed food (25%) and broiler meat (12%). Protection of European consumers from foodborne *Salmonella* infection relies on an integrated approach of food safety from farm to fork (EFSA, https://www.efsa.europa.eu/en/topics/topic/salmonella). Given the role of the poultry reservoir in human salmonellosis, Regulation (EC) No 2160/200317 and its subsequent amendments require that all Member States (MSs) set up national control programmes (NCPs) to reduce the prevalence of the *Salmonella* serovars of public health importance in breeder, laying hen, broiler and turkey flocks. The NCPs rely on regular testing of poultry flocks based on environmental sampling for bacteriological detection of the *Salmonella* serovars of public health importance (i.e., ‘target’ serovars). The serovars of public health importance are the most frequent *Salmonella* causing human salmonellosis and present in the poultry reservoirs (Regulation (EC) No 2160/200317). The European list of targeted serovars in poultry has been established in 2010 (Regulation (EC) No 517/2011). Laying hen flocks detected as infected by a target serovar are culled and/or eggs are destroyed or heat‐treated to destroy *Salmonella* in the final products. Poultry houses are cleaned and disinfected thoroughly before housing the next flock.

Following the coming into force of Regulation (EC) No 2160/200317, the French NCP adopted in 1998 was revised in 2013 and in 2018 to comply with the EU requirements. A description of the NCP and a comparison with NCPs of some other MSs was published (ADONIS Consortium 2022). The present description underlines the main specificities of the French NCP in comparison with the general requirement of the EU regulation. Laying hen flocks with more than 250 birds are subject to *Salmonella* surveillance and to management measures in case of infection by a target serovar. Flocks with fewer than 250 laying hens but delivering eggs to an egg grading plant are also covered by the NCP. *Salmonella* target serovars (STS) in laying hen flocks are *S. Enteritidis* , *S. Typhimurium* , variants of *S. Typhimurium* and *S*. Kentucky (since 2015 for the latter). Three variants of *S. Typhimurium* are controlled in France: 1,4,[5],12:i:‐ (antigenic formula), 1,4,[5],12,i:‐, 1,4,[5],12,‐:1,2 and 1,4,[5],12,‐:‐:. Official sampling carried out by food business operators (FBOs) takes place every 15 weeks during the period of production of the layer flock (Regulation (EC) No 2160/200317). It includes faeces samples (pools of faeces, swabs or boot swabs) and environmental samples (swabs) from dusty surfaces. One pooled sample consisting of 2 × 150 g of fresh faeces or of four swabs applied on scraper belts is taken from cage production systems. In on‐floor production systems, the faecal sample is composed of two pairs of bootswabs, taken from the slatted and the litter areas and analysed as a single pool. Environmental sampling is mandatory for flocks over 1000 hens. The number of environmental swabs taken depends on the number of hens in the flock, ranging from no swab in flocks with less than 1000 birds to four swabs in flocks over 80,000 birds. Swabs for faecal and environmental sampling are premoistened fabric swabs of at least 900 cm^2^. Up to 2018, confirmation sampling was carried out in laying hen flocks detected as infected by a target serovar, sometimes leading to rejection of the suspected infection. Since 2018, confirmation sampling has been limited to circumstances where there is a doubt about the first analysis results. Sampling by the competent authority (CA) is programmed according to Regulation (EU) No 517/2011 requirements. The CA controls one flock per year and per holding housing more than 1000 hens and flocks housed in holdings where the previous flock was detected as positive for STS. Two dust samples (swabs) are taken for CA testing in addition to five faecal samples (pools of faeces, swabs of faeces or bootswabs). Until 2023, vaccination of laying hen flocks for *S*. *Enteritidis* and *S. Typhimurium* was authorised (but not mandatory) with inactivated vaccines, but restricted to exceptional epidemiological situations when using live vaccines.

The review of the epidemiological trends in human salmonellosis over the last 20 years shows an initial decrease in the number of cases in the EU: the number of cases dropped by almost one half between 2005 and 2009 (EFSA, 2011 – https://www.efsa.europa.eu/en/topics/topic/salmonella). This reduction was partly attributed to the implementation of NCPs in poultry flocks (EFSA and ECDC 2011). This decrease in human salmonellosis cases was also observed in France, albeit slightly earlier than in other MSs, occurring as early as the 1999–2003 period. This initial decrease was attributed to the implementation of the first NCP in France, set up in 1998 and based on a similar strategy as Regulation (EC) No 2160/200317 (Poirier et al. 2006). However, the number of salmonellosis cases stopped declining in the EU and in France in 2016 (EFSA and ECDC 2019). From then on, it has plateaued in most MSs, despite a conjectural drop due to the COVID‐19 pandemic in 2020 (EFSA and ECDC 2024). Similarly, stabilisation and even an increase in *Salmonella* prevalence has been observed in poultry flocks in certain MSs since 2016 (EFSA and ECDC 2024). Thus, the ADONIS research project (assessing determinants of the non‐decreasing incidence of *Salmonella*), as part of the One Health European Joint Programme (EJP) (https://onehealthejp.eu/), was launched to identify the determinants linked to this stabilisation and reversal of human salmonellosis incidence in Europe. As a part of the project, results from NCPs were studied in participating countries to identify factors that may be associated with the current change in *Salmonella* epidemiology in poultry flocks. Here, we present the results of *Salmonella* surveillance in laying flocks in France over almost a decade. Our study focused on layer flocks, because eggs remain the main vehicle for *Salmonella* from poultry to humans. The ultimate objective was to propose improvements for the *Salmonella* surveillance system in laying hen flocks.

## 2 Materials & Methods

### 2.1 Data Description

**FIGURE 1:**
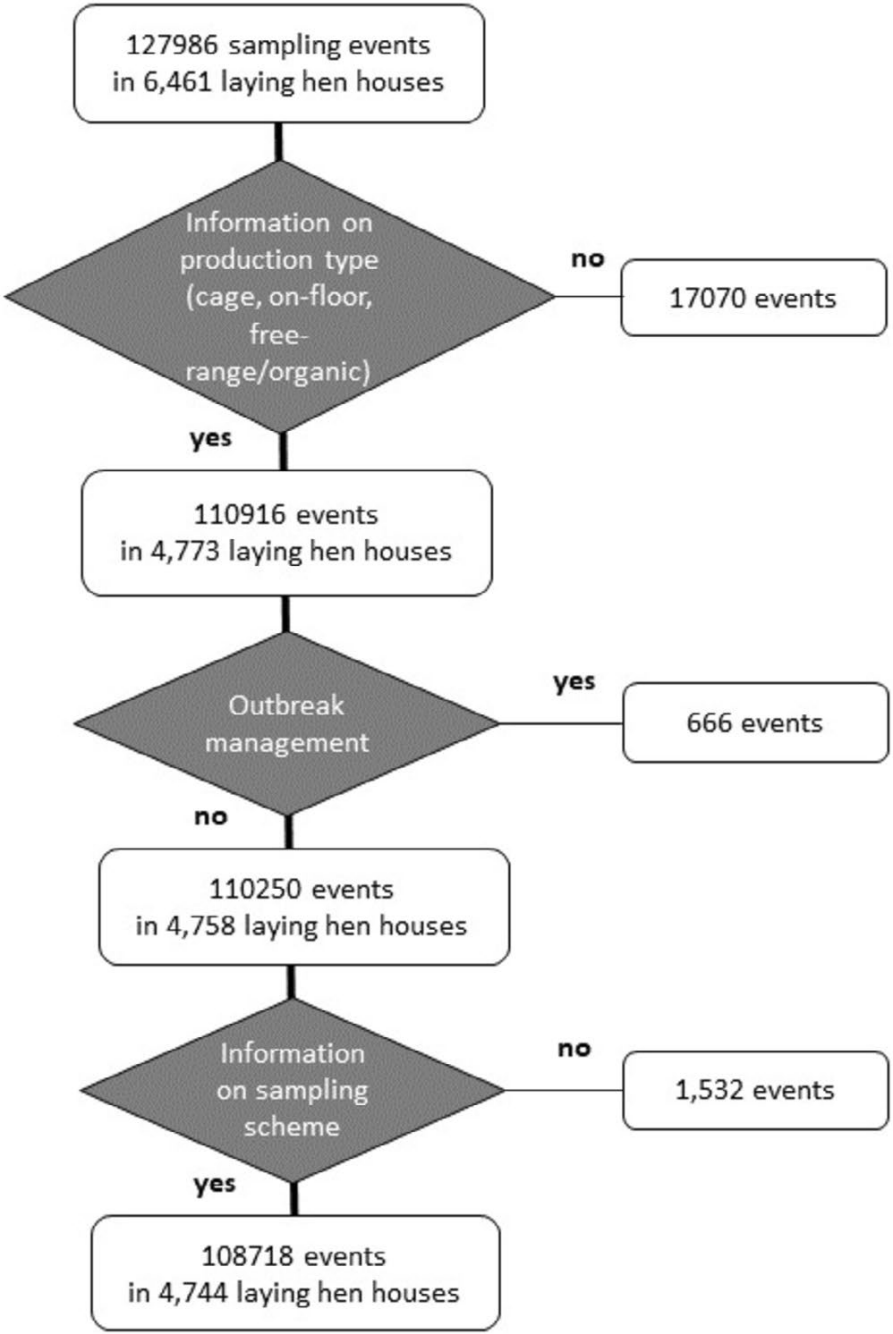
Flow chart of the data processing (*N* = 127,986 sampling events, France, 2013–2021).

Data were obtained from the French Ministry of Agriculture via the National Animal Health Surveillance Platform (https://plateforme‐esa.fr/fr). Results from the *Salmonella* testing in layer flocks are automatically transmitted from the laboratory information management systems of the approved laboratories to a database managed by the Ministry. The current database was implemented in 2011 and definitively consolidated in 2013; no data are available prior to 2013. Automatic transmission of *Salmonella* results is fully implemented for laboratories in mainland France only. Controls for laying hen flocks in overseas French departments were not considered in this study. The study was carried out using data from 127,986 sampling events taking place in 6461 laying hen houses from 01/01/2013 to 31/12/2021. Figure 1 shows the data flow chart describing the data processing. *Salmonella* results were transmitted along with information on sampling context at two levels. The first level of information involved the poultry house: unique poultry house identifier, location (region, municipality) and type of production (cage, on‐floor, free‐range, organic). The latter information was obtained by crosschecking the information in the *Salmonella* database with the information from another database managed by the French Ministry of Agriculture on egg‐marking codes, because the same poultry house identifier is used in both databases. Nevertheless, information on the type of production was missing for 1910 (out of 6461, 30%) poultry houses, accounting for 13% of the sampling events (17,070/127,986). The second level of information involved the sampling procedure and its context: sampler (official sampling performed by the competent authorityor samples taken by FBOs), date of sampling, number of samples and sample matrix (swabs, boot swabs, faeces, dust). Samples taken by the CA for foodborne outbreak investigations or for *Salmonella* infection management on a farm and samples without information on the sampling scheme used were excluded. Missing data on the type and the numbers of samples taken led to the exclusion of 1532 sampling events (out of 110,250). The final database consisted of 108,718 sampling events carried out in 4744 poultry houses. The number of samples taken per poultry house ranged from 1 to 88 (average 19.8 ± 15.3). No information on the flock, such as size, age of the hens at the time of sampling or *Salmonella* vaccination status, was available.

## 3 Statistical Analysis

The outcome of interest is the detection of an STS in at least one of the samples obtained during a sampling event carried out in a laying hen poultry house. Detection of other *Salmonella* serovars was not studied, because reporting of positive results for non‐target serovars is mandatory for controls carried out before slaughter only. It is of note that the frequency of positive controls for STS is lower than the prevalence of STS infection in laying hen flocks as reported to EC and EFSA. This difference is due to the calculation of the frequency of positive STS sampling being relative to the sampling events, whereas prevalence is calculated at the flock level. A laying hen flock can be tested on several occasions (negative tests) before being detected as positive.

The probability of a sampling being positive for STS was described in terms of frequency distribution (using R 4.1.2, R Core Team 2023). Crude odds ratios (ORs) were calculated for explanatory variables at the poultry house level and the sampling level. Associations between explanatory variables were also evaluated based on chi squared tests and Cramer's V measure. In case of strong association between two explanatory variables, only the variable the most associated with the outcome was kept for further analysis. A mixed logistic regression model was fitted taking into account repeated sampling at the poultry house level using a ‘poultry house’ random intercept (function glmer, package lme4, Bates et al. 2015). The general equation of the model was

$$\log\left(\frac{p_{\mathrm{ij}}}{1-p_{\mathrm{ij}}}\right)=\beta_{0}+b_{i}+\beta_{1}X_{i}+e_{\mathrm{ij}}$$

where, $p_{\mathrm{ij}}$, denotes the probability of obtaining a positive STS result for the *j*th sampling in the *i*th poultry house. $\beta_{0}$, denotes the fixed intercept. $b_{i}$, denotes the random intercept for the *i*th poultry house. $\beta_{1}$, denotes the fixed‐effect parameter vector associated with $X_{i.}$ $X_{\mathrm{ij}}$, denotes the vector of predictors, that is, explanatory variables. $e_{\mathrm{ij}}$, denotes the error of the model.

Variables for which evidence of association was not found (e.g., 95% probability intervals of the estimated ORs including 1) were excluded during the back–forward procedure. Interactions in the final model were tested and removed if not significant at the *p* = 0.05 level. Multicollinearity between predictors in the final model was checked by calculating the variance inflation factor (VIF) associated with explanatory variables (function check_collinearity, package performance, Lüdecke et al. 2021). Introducing the poultry house effect as a random effect made it possible to draw conclusions on the fixed effects, taking into account the potential effect of the poultry house. Generalisation of the results was easier, but no conclusions could be drawn at the individual house level. The risk of *Salmonella* recurrence in the same house was taken into account but not quantified.

## 4 Results

**FIGURE 2:**
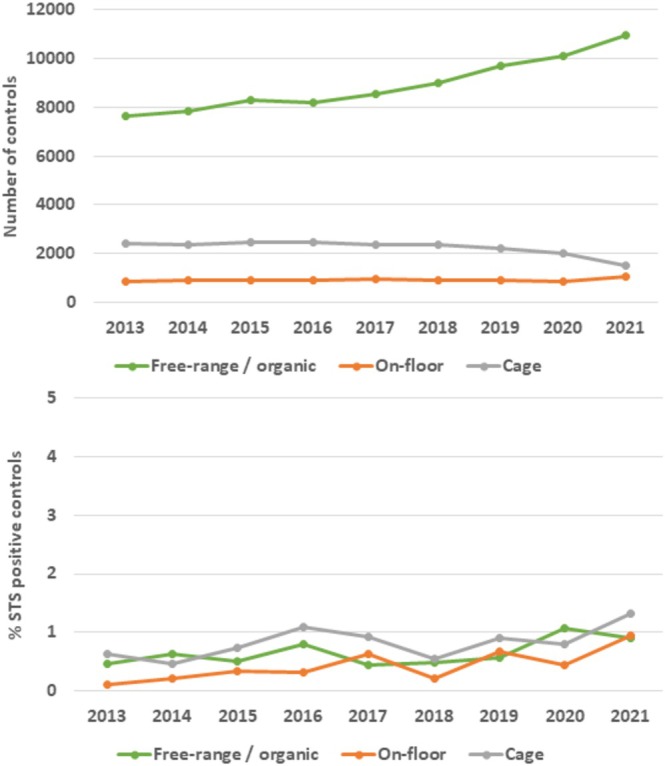
Number of sampling events *(top)* and frequencies *(bottom)* of sampling events that detected a *Salmonella* target serovar (STS) according to type of production in laying hen poultry houses (*N* = 108,718 sampling events as part of the national control programme, France, 2013–2021).

**FIGURE 3:**
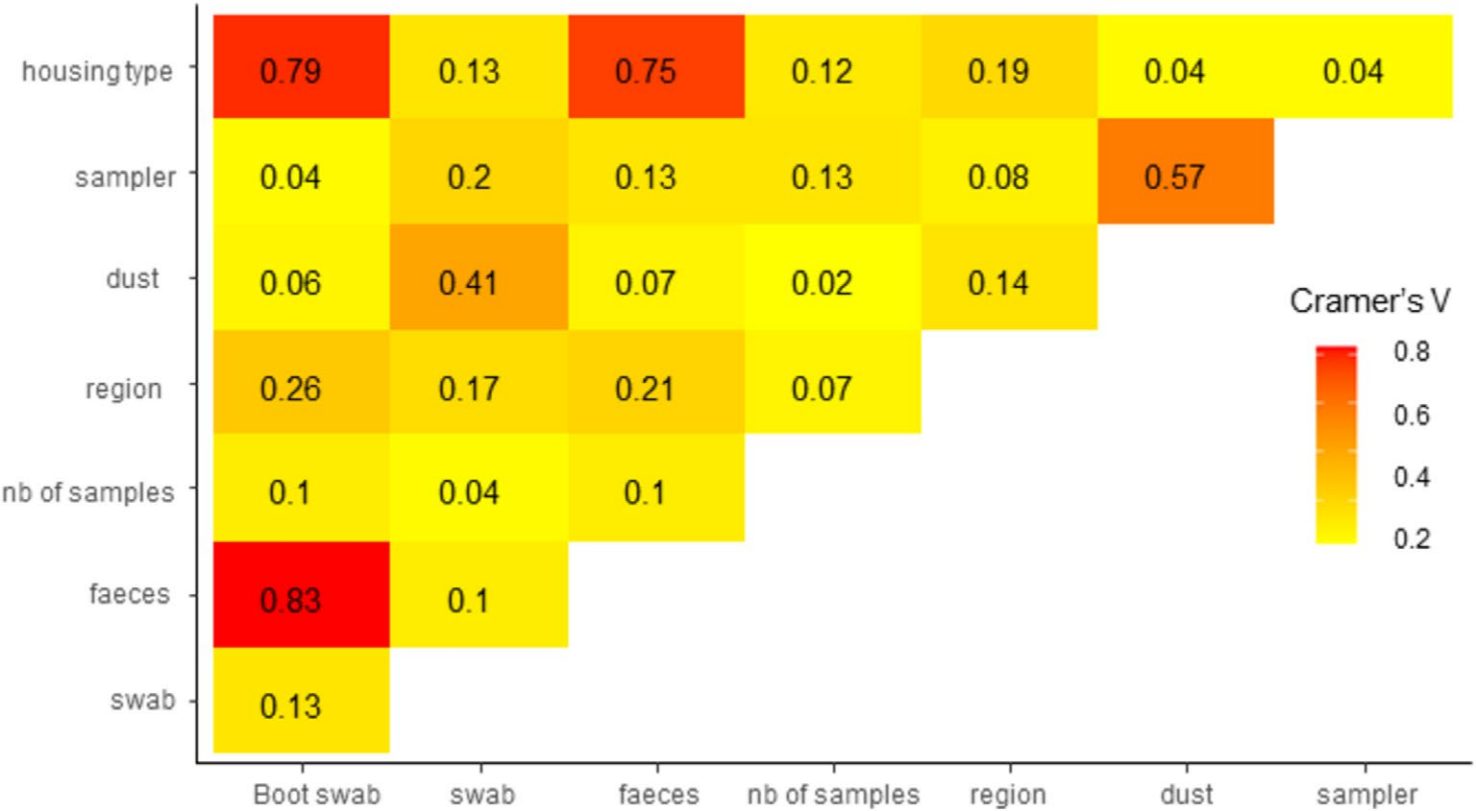
Matrix of Cramer's V association index for explanatory variables at the poultry house level and the sampling level obtained from the *Salmonella* national control programme database in laying hen houses (*N* = 108,708 sampling events, France, 2013–2021).

STS were isolated from 737 sampling events out of 108,718 (0.68%) and 621 poultry houses (out of 4744, 13%) showed a positive sampling at least once over the 9‐year study period. Up to four positive sample events were observed in one poultry house. The relative risk of a sampling event being STS‐positive when the house previously housed a positive flock was 2.4 (IC_95%_ [2.0–2.9]). *S. Enteritidis* was the most frequently detected STS (453 positive sampling events, 61%), followed by *S. Typhimurium* (235, 32%), *S. Typhimurium* monophasic variants (59, 8%) and *S*. Kentucky (10, 1%). Multiple STS were isolated from 20 sample events (2.6% of the STS‐positive sampling events). No statistical association was observed between the types of positive STS samples (faecal sample, swabs, bootswabs or dust) and the serovar isolated (data not shown). The frequency of STS‐positive sample events varied from 0.48% in 2013 to 0.99% in 2020 (Table 1). The number of sampling events per year has increased in organic production and free‐range production systems since 2017, but decreased for cage production systems over the same period (Figure 2). The risk of an STS‐positive sampling event was higher in cage production (OR = 1.2 [1.0–1.4]) relative to free‐range or organic production (Table 1). The risk was the lowest for on‐floor production without an open‐air range (OR = 0.67, [0.47–0.92]). Sampling events carried out by the CA represented 16% of the sampling events, but accounted for 64% of the events that detected an STS. Consequently, the risk of an STS‐positive sampling was higher when samples were taken by the CA (OR = 3.1 [2.6–3.6]) than by FBOs. The number and the type of samples taken also influenced the probability of an STS‐positive sampling. Types of samples taken varied with the type of production (Figure 3); variables on types of sample were not introduced in the final model.

**TABLE 1:** Results of target *Salmonella* testing and associated odds ratios (OR) according to poultry house and sampling event characteristics in laying hen poultry houses (National Control Programme, 108,718 sampling events, France, 2013–2021).

Variable		Number of sampling events	Negative sampling events	STS^a^ positive sampling events	% Positive	Crude odd ratio 95% CI^b^	*p*
Type of housing	On‐floor	8260	8223	37	0.45	0.67 [0.47–0.92]	0.02
	Cage	20,134	19,972	162	0.80	1.2 [1.0–1.4]	0.04
	Free‐range/organic	80,324	79,786	538	0.67	1	
Region	South‐East	16,737	16,496	241	1.4	3.8 [3.1–4.7]	< 0.001
	South	5453	5384	69	1.3	3.3 [2.5–4.5]	< 0.001
	East	8615	8532	83	0.96	2.5 [1.9–3.3]	< 0.001
	South‐West	6339	6293	46	0.73	1.9 [1.3–2.6]	< 0.001
	North	11,099	11,037	62	0.56	1.5 [1.1–2.0]	0.02
	Centre	9215	9175	40	0.43	1.1 [0.8–1.6]	0.49
	Centre‐West	19,962	19,866	76	0.38	1.0 [0.7–1.3]	0.97
	West	31,318	31,198	120	0.38	1	
Year	2013	10,903	10,851	52	0.48	1	
	2014	11,129	11,066	63	0.57	0.67 [0.47–0.92]	0.36
	2015	11,614	11,551	63	0.54	1.2 [0.82–1.7]	0.49
	2016	11,598	11,502	96	0.83	1.7 [1.2–2.5]	0.001
	2017	11,865	11,799	66	0.56	1.2 [0.81–1.7]	0.40
	2018	12,268	12,210	58	0.47	1.0 [0.68–1.4]	0.96
	2019	12,801	12,719	82	0.64	1.3 [0.95–1.9]	0.09
	2020	13,006	12,877	129	0.99	2.1 [1.5–2.9]	< 0.001
	2021	13,534	13,406	128	0.95	2.0 [1.4–2.8]	< 0.001
Quarter	Jan–Mar	27,059	26,953	106	0.39	1	
	Apr–Jun	27,428	27,341	87	0.32	0.81 [0.61–1.1]	0.14
	Jul–Sept	26,907	26,614	293	1.1	2.8 [2.2–3.5]	< 0.001
	Oct–Dec	27,324	27,073	251	0.92	2.4 [1.9–3.0]	< 0.001
Sampler	Competent authority	16,950	16,684	266	1.6	3.1 [2.6–3.6]	< 0.001
	Food Business Operator	91,768	91,297	471	0.51	1	
Number of samples	6 or more	1620	1566	54	3.3	5.4 [4.0–7.0]	< 0.001
	[1–5]	107,098	106,415	683	0.64	1	
Dust sample	Yes	6559	6478	81	1.2	1.9 [1.5–2.4]	< 0.001
	No	102,159	101,503	656	0.64	1	
Faeces sample	Yes	19,180	19,075	105	0.55	0.77 [0.63–0.95]	0.02
	No	89,538	88,906	632	0.71		
Boot swab	Yes	86,591	86,019	572	0.66	0.89 [0.75–1.6]	0.17
	No	22,127	21,962	165	0.75	1	
Swab	Yes	98,782	98,143	639	0.65	0.65 [0.53–0.81]	< 0.001
	No	9936	9838	98	0.99	1	

^a^ *Salmonella* Targeted Serovar.

^b^ Confidence interval.

**FIGURE 4:**
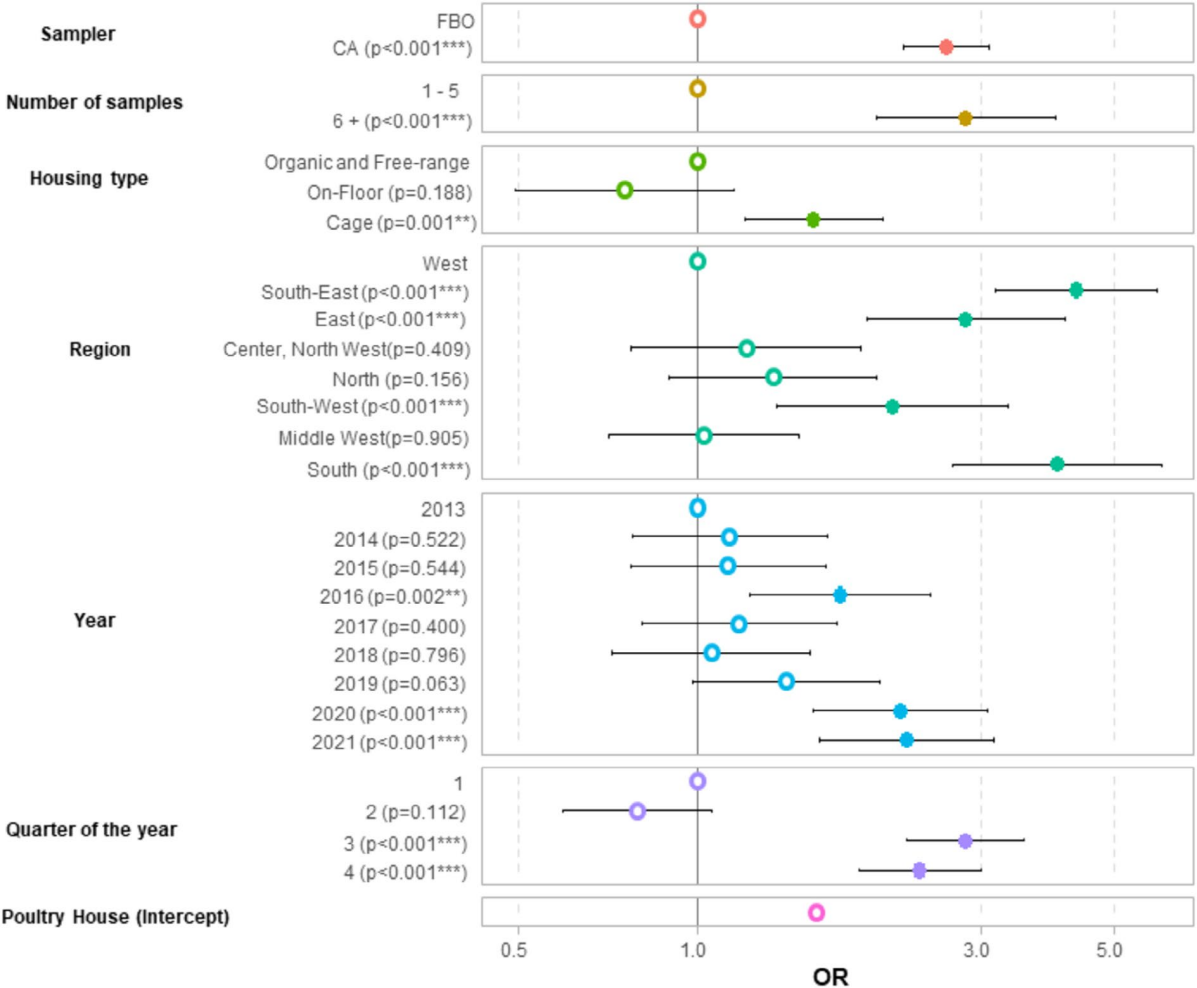
Variables associated with an increased risk of *Salmonella* target serovars detection in controls conducted in the framework of the national control programme in laying hen poultry houses, according to a mixed‐model logistic regression (*N* = 108,718 sampling events, France, 2013–2021).

**FIGURE 5:**
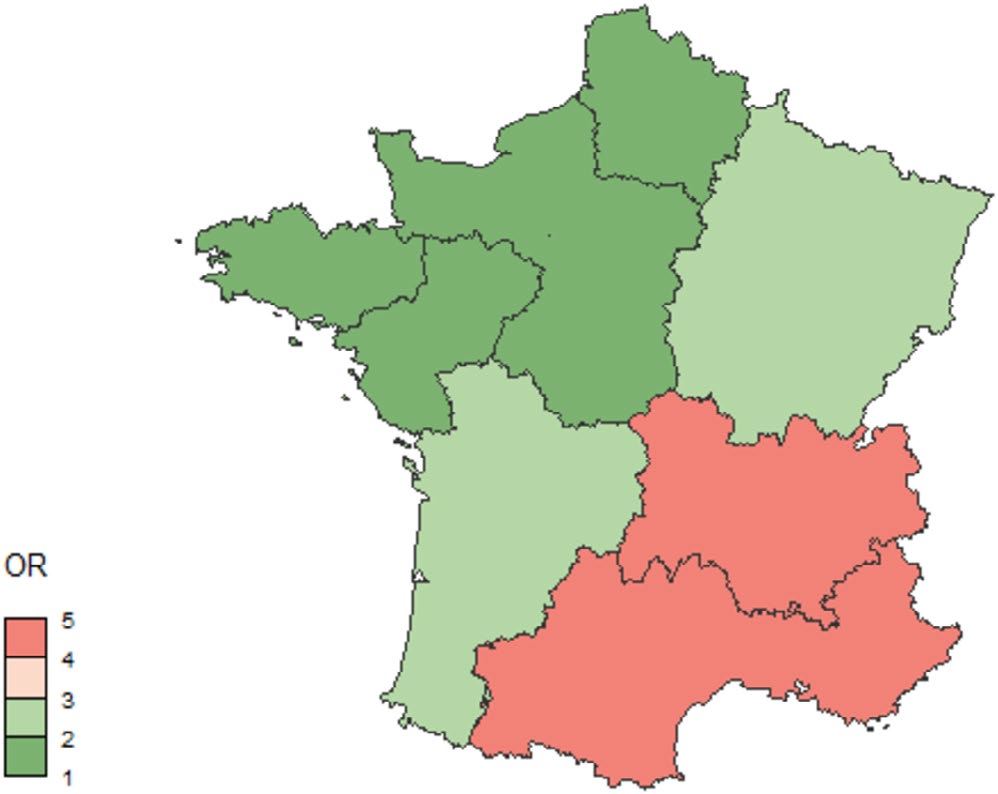
Regional adjusted Odds Ratios for the risk of *Salmonella* target serovars detection in controls conducted as part of the national control programme in laying hen poultry houses, according to a mixed‐model logistic regression (*N* = 108,718 sampling events, France, 2013–2021).

The final model (Figure 4) included variables at the poultry house (location, production type) and sampling levels (sampler, quarter and year of sampling). The complete model estimates are presented in Table S1. Tables S2 and S3 show the results for SE and ST separately. STS detection was related to the administrative region, with higher risks in the East and South‐East regions relative to the West region, used as the reference (Figure 5). Similarly, a higher risk of STS detection was observed for sampling events conducted in the cage production system compared with on‐floor, free‐range or organic production systems. At the sampling level, the risks of detecting STS were much higher when sampling was conducted by the CA, when more than 5 samples were taken and in the second half of the year (from July to December). Certain years were marked by a higher risk of STS detection, that is, 2016, 2020 and 2021.

## 5 Discussion

This work is the first to study the results of the NCP in French laying hen farms since the implementation of the EU regulation in 2013. France is one of the main egg producers in the European Union with 1570 billion eggs in 2021 (ITAVI 2023). The French egg sector has grown rapidly since 2016, with increases in the number of producing farms and in the proportion of farms in on‐floor, free‐range, and organic systems. These trends were clear in the results of the NCP from 2013 to 2021. The present study also underlined the increase in the frequency of STS‐positive sampling events, increasing from 0.48% in 2013 to 0.99% in 2020. A similar trend has been reported at the EU level, but for all *Salmonella* serotypes (EU Zoonosis) and in Spain (Samper‐Cativiela et al. 2023). No link could be established between the increase in STS‐positive sampling events and the development of free‐range and organic laying hen farms observed during the 2016–2021 period. Our results show that the proportion of positive sampling events increased in all production systems (not only in open‐air systems) from 2019 to 2021. In addition, the cage production system was the most at‐risk system for STS detection (OR = 1.56 [1.20–2.05], relative to free‐range and organic production systems), as previously observed in the *Salmonella* baseline study in France in 2003 (Huneau‐Salaün et al. 2009) or more recently in Spain (Samper‐Cativiela et al. 2023). Geographical variability was also observed in frequencies of positive controls according to regions (the largest administrative division in France). This spatial heterogeneity was expected based on annual results from the NCP programme (Huneau‐Salaün et al. 2021), but had not been precisely described and quantified to date. Dedicated epidemiological surveys including more information on farm management and local characteristics are needed to better mitigate *Salmonella* contamination in the at‐risk areas.

An increase in STS detection was observed from 2019 to 2021. A revision in the French NCP occurred in 2018 consisting of the restriction of confirmation sampling after a first STS positive sampling event. Before 2019, all flocks detected positive for STS were systematically retested. Confirmation sampling led to rejection of about 30% of the STS‐positive sampling events before 2018 (Huneau‐Salaün et al. 2021). Because of the restriction of confirmation sampling, most of the STS‐positive sampling events were directly considered as official STS outbreaks after 2018, increasing the total number of outbreaks per year. This increase was expected after the NCP revision but not to such an extent: considering the average frequency of STS‐positive sampling over the 2013–2018 period (0.43%), the average frequency of STS‐positive sampling should be approximately 0.57% per year after 2018 (considering an increase of 33% due the ban of reconfirmation sampling) but the average frequency observed was 0.66% over the 2018–2021 period). The increase may also be due to a slackening of *Salmonella* control measures on farms or to the establishment of new laying hen farms with farmers less experienced in *Salmonella* control. We did not have any information to confirm these hypotheses directly. However, an analysis of epidemiological surveys carried out in *Salmonella* outbreaks in 2020 showed that the main identified risks for *Salmonella* introduction on laying hen farms were poor rodent control measures and flaws in the biosecurity measures on the farm (Huneau‐Salaün et al. 2022). The CA is currently carrying out several actions to evaluate the biosecurity measures on poultry farms to better control this potential issue in *Salmonella* and avian influenza prevention. Another action is the relaxation of French legislation on *Salmonella* vaccination regarding the use of attenuated live vaccines in laying hen farms. The authorisation for using live *Salmonella* vaccines (strictly restricted until 2023) has been expanded to all laying hen farms complying with the biosecurity requirements set by the French regulations for *Salmonella* and avian influenza control. The change in the legislation may increase the proportion of vaccinated flocks in the next years. However, no epidemiological indicator of vaccination coverage has been calculated at the national level, limiting our capacity to evaluate the impact of the change in vaccination regulation on *Salmonella* prevalence.

Several factors may explain the higher risk of detecting an STS‐associated with sampling by the CA, also reported in the United Kingdom (Arnold et al. 2023) and at the EU level for broiler carcasses (EFSA and ECDC 2024). Firstly, part of the sampling events performed by the CA consists of risk‐based sampling, whereas FBOs perform routine monitoring only (Regulation (EU) No 517/2011). To limit this bias, we did not consider sampling events carried out by the CA for epidemiological surveys (follow‐up controls in poultry houses linked to an outbreak) or triggered by a suspicion of STS infection in a flock or by human cases of salmonellosis linked to the flock. We considered CA testing planned on an annual basis or in flock held in a previously contaminated poultry house. Nevertheless, some of these sampling events are still part of risk‐based sampling because they target poultry houses where an STS infection occurred previously. This targeting may increase the risk of obtaining a STS detection but we could not estimate the proportion of risk‐based sampling events in the database (no information available). Secondly, testing carried out by the CA accounted for more samples than those carried out by the FBOs. In addition, more types of sample (boot swabs, swabs, faecal sample or dust) were taken by the CA than by FBOs. For example, 37% of the sampling events done by the CA included one or more dust samples, but this type of sample was taken only in 0.2% of the sampling events by FBOs. Previous studies showed that the sensitivity of the sampling scheme for *Salmonella* detection in a laying hen flock varies according to the number of samples taken and the type of sample (Mahé et al. 2007; Arnold et al. 2013). More specifically, dust sampling is more sensitive than faeces sampling for detecting *Salmonella* in cage production systems. The sampling schemes used by the CA may be more sensitive than the routine sampling schemes used by FBOs. This was the case in the United Kingdom where the CA sampling including a dust sample was five more time sensitive than the FBOs sampling based on faecal samples only (Arnold et al. 2023). For FBOs in France, environmental samples (swabs) are taken for flocks with more than 1000 laying hens. This is a special provision of the French NCP, not required in Regulation (EU) No 517/2011. Adding environmental sampling aims to improve the sensitivity of the sampling scheme in comparison with sampling schemes including faecal samples only. The number of environmental samples is also correlated with flock size: the mandatory numbers of swabs to be taken increases by one for every 20,000 laying hens, up to four samples in flocks over 80,000 birds, following the requirements of French regulations. Large flock size is a risk factor for *Salmonella* infection in laying hens (Snow et al. 2010; Huneau‐Salaün et al. 2009). In addition, large flocks are more common in cage production system than in on‐floor, free‐range or organic systems. It is therefore difficult to determine the respective impact of those three factors on the risk of infection.

Our study relied on an exhaustive collection of NCP results (for mainland France), limiting potential biases of selection and improving statistical power for risk factor identification. The 9‐year study period made it possible to observe epidemiological trends at the national level. Nevertheless, the automatic data collection process limits the range of epidemiological data collected to basic sample description and poultry house identification. In particular, the data description and analysis were limited to the sampling event level, because no flock identification system is in place. Therefore, no direct comparison with EU prevalence data can be carried out. In addition, the lack of information at the flock level limits the epidemiological conclusions that can be drawn. For example, the impact of *Salmonella* vaccination (used by farmers on a voluntary basis) cannot be evaluated, because no information on vaccine use is available in the NCP database. In 2023, a working group (under the National animal health surveillance Platform) involving FBOs, the CA, and scientists, was launched to improve the quality and completeness of the epidemiological information collected in the *Salmonella* NCP in France (Huneau‐Salaün et al. 2025).

We carried out the first analysis of NCP results in laying hen farms in France since the implementation of the EU Regulation in 2013. Our results underlined the trend of the increasing frequency of *Salmonella*‐positive sampling events observed since 2018 in France. They also highlighted the geographical heterogeneity of the *Salmonella* contamination risk on farms in France. Sampling events carried out by the CA were more frequently *Salmonella*‐positive than those carried out by FBOs, due to the risk‐based sampling applied by the CA and a high number of samples taken. In addition, the risk of detecting *Salmonella* was associated with the type and the number of samples taken. Our findings are of interest for triggering prevention actions specific to at‐risk areas and for guiding the development of improved sampling protocols for *Salmonella* detection in layer farms in France.

## Ethics Statement

The authors have nothing to report.

## Consent

The authors have nothing to report.

## Conflicts of Interest

The authors declare no conflicts of interest.

## Supporting information


**Table S1.** Mixed logistic regression model for the risk of a positive a *Salmonella* target serovar (STS) sampling event in laying hen poultry houses (national control programme, 108,718 sampling events, France, 2013–2021).
**Table S2.** Mixed logistic regression model for the risk of a positive a *Salmonella enteritidis* sampling event in laying hen poultry houses (national control programme, 108,718 sampling events, France, 2013–2021).
**Table S3.** Mixed logistic regression model for the risk of a positive a *Salmonella typhimurium* (and its variants) sampling event in laying hen poultry houses (national control programme, 108,718 sampling events, France, 2013–2021).

## Data Availability

The data that support the findings of this study are available from Direction Générale de l'Alimentation. Restrictions apply to the availability of these data, which were used under license for this study.
