# Supplementary material for: Salmonella Control Programme in France: Factors Influencing the Detection of Salmonella in Laying Hen Flocks From 2013 to 2021
Source: Zoonoses Public Health. 2025 Jul 16;72(7):628–36. doi: 10.1111/zph.70001 (PMC12508787; doi:10.1111/zph.70001)
Supplement: Supplementary file 1 — Table S1. Mixed logistic regression model for the risk of a positive a Salmonella target serovar (STS) sampling event in laying hen poultry houses (national control programme, 108,718 sampling events, France, 2013–2021). Table S2. Mixed logistic regression model for the risk of a positive a Salmonella enteritidis sampling event in laying hen poultry houses (national control programme, 108,718 sampling events, France, 2013–2021). Table S3. Mixed logistic regression model for the risk of a positive a Salmonella typhimurium (and its variants) sampling event in laying hen poultry houses (national control programme, 108,718 sampling events, France, 2013–2021). [file ZPH-72-628-s001.docx]

Table S1: Mixed logistic regression model for the risk of a positive a *Salmonella* target serovar (STS) sampling event in laying hen poultry houses (National Control Programme, 108718 sampling events, France, 2013–2021)

| Predictors | Odds Ratio | CI† | P |
| --- | --- | --- | --- |
| Poultry House Intercept | 0.00 | 0.00 – 0.00 | **<0.001** |
| Type of Production System  On-Floor  Cage  Organic / Free-Range | 0.75  1.56  1 | 0.49 – 1.15  1.20 – 2.05 | 0.188  **0.001** |
| Region  South-East  South  East  South-West  North  Centre  Centre-West  West | 4.32  4.02  2.82  2.12  1.34  1.21  1.02  1 | 3.16 – 5.91  2.69 – 6.02  1.92 – 4.14  1.36 – 3.32  0.89 – 2.00  0.77 – 1.88  0.71 – 1.48 | **<0.001**  **<0.001**  **<0.001**  **0.001**  0.156  0.409  0.905 |
| Year  2013  2014  2015  2016  2017  2018  2019  2020  2021 | 1  1.13  1.12  1.73  1.17  1.05  1.41  2.19  2.24 | 0.78 – 1.65  0.77 – 1.64  1.22 – 2.46  0.81 – 1.71  0.72 – 1.55  0.98 – 2.02  1.57 – 3.07  1.60 – 3.14 | 0.522  0.544  **0.002**  0.400  0.796  0.063  **<0.001**  **<0.001** |
| Quarter of the year  Jan-Mar  Apr-Jun  Jul-Sep  Oct-Dec | 1  0.79  2.82  2.36 | 0.59 – 1.06  2.24 – 3.54  1.87 – 2.99 | 0.112  **<0.001**  **<0.001** |
| Sampler  Competent Authority  Food Business Operators | 2.62  1 | 2.22 – 3.10 | **<0.001** |
| Number of samples  1 to 5  6 or more | 1  2.82 | 1.99 – 4.00 | **<0.001** |
| **Random Effects** | | | |
| σ^2^ | 3.29 | | |
| τ_00_ _house_ ^‡^ | 2.52 | | |
| Intra-Class Correlation | 0.43 | | |
| N _house_ | 4744 | | |
| Observations | 108718 | | |
| Marginal R^2^ / Conditional R^2^ | 0.135 / 0.510 | | |

^†^ 95% Confidence Interval

^‡^ Standard errors of the random poultry-house effect

Table S2: Mixed logistic regression model for the risk of a positive a *Salmonella* Enteritidis sampling event in laying hen poultry houses (National Control Programme, 108718 sampling events, France, 2013–2021)

| Predictors | Odds Ratio | CI† | P |
| --- | --- | --- | --- |
| Poultry House Intercept | 0.00 | 0.00 – 0.00 | **<0.001** |
| Type of Production System  On-Floor  Cage  Organic / Free-Range | 0.91  1.35  1 | 0.45 – 1.84  0.84 – 2.14 | 0.78  0.21 |
| Region  South-East  South  East  South-West  North  Centre  Centre-West  West | 6.23  4.12  2.16  1.94  0.98  0.78  0.94  1 | 4.12 - 9.42  2.21 – 7.68  1.15 – 4.04  1.93 – 1.95  0.48 – 2.00  0.33 – 1.83  0.52 – 1.70 | **<0.001**  **<0.001**  **0.02**  **<0.001**  0.95  0.56  0.84 |
| Year  2013  2014  2015  2016  2017  2018  2019  2020  2021 | 1  1.27  1.37  2.37  1.62  0.93  1.67  2.33  3.53 | 1.26 – 1.27  0.92 – 2.03  1.67 – 3.36  0.10 – 2.38  0.58 – 1.47  0.14 – 2.43  1.65 – 3.30  2.56 – 4.87 | **<0.001**  0.12  **<0.001**  **0.01**  0.74  **0.001**  **<0.001**  **<0.001** |
| Quarter of the year  Jan-Mar  Apr-Jun  Jul-Sep  Oct-Dec | 1  1.03  3.97  3.30 | 1.02 - 1.03  3.25 – 4.86  3.28 – 3.32 | **<0.001**  **<0.001**  **<0.001** |
| Sampler  Competent Authority  Food Business Operators | 2.88  1 | 2.31-3.59 | **<0.001** |
| Number of samples  1 to 5  6 or more | 1  2.74 | 1.69 – 4.43 | **<0.001** |
| **Random Effects** | | | |
| σ^2^ | 3.29 | | |
| τ_00_ _house_ ^‡^ | 14.99 | | |
| Intra-Class Correlation | 0.82 | | |
| N _house_ | 4,744 | | |
| Observations | 108,718 | | |
| Marginal R^2^ / Conditional R^2^ | 0.067 / 0.832 | | |

^†^ 95% Confidence Interval

^‡^ Standard errors of the random poultry-house effect

Table S3: Mixed logistic regression model for the risk of a positive a *Salmonella* Typhimurium (and its variants) sampling event in laying hen poultry houses (National Control Programme, 108718 sampling events, France, 2013–2021)

| Predictors | Odds Ratio | CI† | P |
| --- | --- | --- | --- |
| Poultry House Intercept | 0.00 | 0.00 – 0.00 | **<0.001** |
| Type of Production System  On-Floor  Cage  Organic / Free-Range | 0.42  1.25  1 | 0.13 – 1.38  0.74 – 2.14 | 0.15  0.41 |
| Region  South-East  South  East  South-West  North  Centre  Centre-West  West | 0.71  2.17  1.41  0.98  0.75  0.81  0.48  1 | 0.38 – 1.45  2.16 – 2.18  0.68 – 2.94  0.37 – 2.56  0.34 – 1.94  0.34 – 1.94  0.21 – 1.12 | 0.38  **<0.001**  0.35  0.96  0.64  0.64  0.09 |
| Year  2013  2014  2015  2016  2017  2018  2019  2020  2021 | 1  0.47  0.60  0.62  0.44  0.63  0.75  1.24  0.70 | 0.29 – 0.76  0.60 – 0.60  0.61 – 0.62  0.27 – 0.82  0.41 – 0.97  0.50 – 1.13  0.87 – 1.76  0.46 – 1.08 | **0.002**  **<0.001**  **<0.001**  **0.001**  **0.03**  0.16  0.24  0.11 |
| Quarter of the year  Jan-Mar  Apr-Jun  Jul-Sep  Oct-Dec | 1  0.57  2.30  1.98 | 0.56 – 0.57  2.29 – 2.31  1.97 – 1.99 | **<0.001**  **<0.001**  **<0.001** |
| Sampler  Competent Authority  Food Business Operators | 2.49  1 | 2.48-2.50 | **<0.001** |
| Number of samples  1 to 5  6 or more | 1  2.71 | 1.47 – 5.02 | **<0.001** |
| **Random Effects** | | | |
| σ^2^ | 3.29 | | |
| τ_00_ _house_ ^‡^ | 14.99 | | |
| Intra-Class Correlation | 0.82 | | |
| N _house_ | 4,744 | | |
| Observations | 108,718 | | |
| Marginal R^2^ / Conditional R^2^ | 0.067 / 0.832 | | |

^†^ 95% Confidence Interval

^‡^ Standard errors of the random poultry-house effect
